# The prognostic value of C-reactive protein/albumin ratio in nasopharyngeal carcinoma: a meta-analysis

**DOI:** 10.1042/BSR20180686

**Published:** 2018-11-14

**Authors:** Nan Gao, Ruo-Nan Yang, Zhen Meng, Wan-Hai Wang

**Affiliations:** Clinical Laboratory, The First Affiliated Hospital of Zhengzhou University, Key Laboratory of Laboratory Medicine of Henan Province, Zhengzhou 450052, Henan, China

**Keywords:** C-reactive protein/albumin ratio, meta analysis, nasopharyngeal carcinoma, prognosis

## Abstract

The C-reactive protein/albumin ratio (CRP/Alb ratio) has been reported to have promising prognostic value in several cancers. The current meta-analysis was conducted to better define the prognostic value of CRP/Alb ratio in patients with nasopharyngeal carcinoma (NPC). The Web of Science, Embase, Cochrane Library databases, and PubMed were searched up to 25 February 2018 for the information on CRP/Alb ratio and outcomes of NPC. Pooled hazard ratios (HRs) and corresponding 95% confidence intervals (95% CIs) were used to evaluate the association between CRP/Alb ratio and survival outcomes in NPC. A total of five studies with 5533 patients with NPC were included. Pooled results showed that high CRP/Alb ratio was associated with poor overall survival (OS) (HR = 1.51, 95% CI: 1.30–1.75, *P*<0.001) and poor distant metastasis-free survival (DMFS) (HR = 1.23, 95% CI: 1.07–1.43, *P*=0.005). Subgroup analyses showed that patients with higher CRP/Alb ratio have worse OS in NPC. In conclusion, elevated CRP/Alb ratio was associated with worse prognosis in patients with NPC.

## Introduction

Nasopharyngeal carcinoma (NPC) has a unique geographic and ethnic distribution, with the highest incidence rates in South-Eastern Asia [1]. NPC is derived from the nasopharynx epithelium and its etiology contains viral infection, genetic and social environmental factors [2]. Because of its special anatomical location and radiosensitivity, radiotherapy is the first choice to treat early stage patients with NPC. In addition, combining chemotherapy with radiotherapy is essential for advanced NPC stage treatment [3]. Over the past several decades, the treatment results have improved because of the advancement of the above treatments [4]. However, distant metastasis and recurrence are still the problems for gaining good overall prognosis in NPC patients. Exploring available and determinable prognostic indices may contribute to optimize the treatment methods for patients with NPC.

Increasing evidence has showed that inflammatory response can promote the development and progression of cancers and affect the survival outcomes of patients with cancer [5]. Therefore, lots of cancer researchers concentrate on the identification of cancer-related inflammation biomarkers. The C-reactive protein/albumin ratio (CRP/Alb ratio) is a novel inflammation-based marker and reported to perform promising prognostic value in hepatocellular carcinoma [6], clear cell renal cell carcinoma [7], small-cell lung cancer [8], esophageal squamous cell carcinoma [9], pancreatic cancer [10,11], and colorectal cancer [12]. Meta-analysis is a systematic quantitative research method to integrate individual research findings. It provides a more comprehensive description of the current status of research in a field and a more convincing assessment of clinical outcomes [13]. Thus, we performed a meta-analysis to explore the association between CRP/Alb ratio and survival outcomes in patients with NPC.

## Materials and methods

### Search strategy

The Web of Science, Embase, Cochrane Library databases, and PubMed were searched up to 25 February 2018 for the information on CRP/Alb ratio and outcomes of NPC. Two reviewers (N.G. and R.-N.Y.) independently assessed the eligibility of the studies. The detailed search strategy used in PubMed was as follows: (‘CRP/Alb ratio’ [All Fields] OR ‘C-reactive protein/albumin ratio’ [All Fields] OR ‘CAR’ [All Fields]) AND (‘Nasopharyngeal cancer’ [All Fields] OR ‘Nasopharyngeal carcinoma’ [All Fields] OR ‘Nasopharyngeal tumor’ [All Fields] OR ‘head and neck cancer’ [All Fields] OR ‘NPC’ [All Fields]). The references within the retrieved articles were further screened to identify additional, potentially relevant studies.

### Inclusion and exclusion criteria

Studies were considered eligible and included if they met all the following criteria: (i) the study was published in English; (ii) the CRP/Alb ratio was evaluated before any treatment; (iii) the pretreatment CRP and Alb levels were measured by serum-based methods; (iv) the prognostic value of CRP/Alb ratio was investigated in NPC; (v) overall survival (OS) was included and hazard ratio (HR) with 95% confidence interval (95% CI) were available or could be extracted from the Kaplan–Meier curve.

The exclusion criteria were as follows: (i) full text not available, meta-analyses, reviews, letters, and duplicates; (ii) no survival outcome included or no usable HR with 95% CI; (iii) nonhuman studies.

### Data extraction and quality assessment

Two investigators (N.G. and R.-N.Y.) retrieved studies independently, and any discrepancies were solved by discussing with a third reviewer (W.-H.W). Extracted relative data included first author, publication year, country, sample size, cancer stage, treatment, cut-off value for CRP/Alb ratio, outcome, HR with 95% CI and follow-up time. The Newcastle–Ottawa Scale (NOS) [14] was used to assess the quality of included studies.

### Statistical analysis

Statistical analysis was conducted using Stata 12.0. Pooled HRs and corresponding 95% CIs were used to evaluate the relation between CRP/Alb ratio and survival outcomes. The heterogeneity amongst studies was tested by the *Q-*test and *I^2^* statistic [15]. When there was significant heterogeneity (*I^2^* > 50% or/and *P*<0.10), a random-effect model was used; otherwise, a fixed-effect model was used. HR > 1 implied a worse prognosis for the high CRP/Alb ratio group and if corresponding 95% CI did not cover 1, there could be statistically significant. Publication bias was evaluated by Begg’s and Egger’s tests [16,17]. Statistical significance was defined as *P*<0.05.

## Results

### Study search information

**Figure 1:**
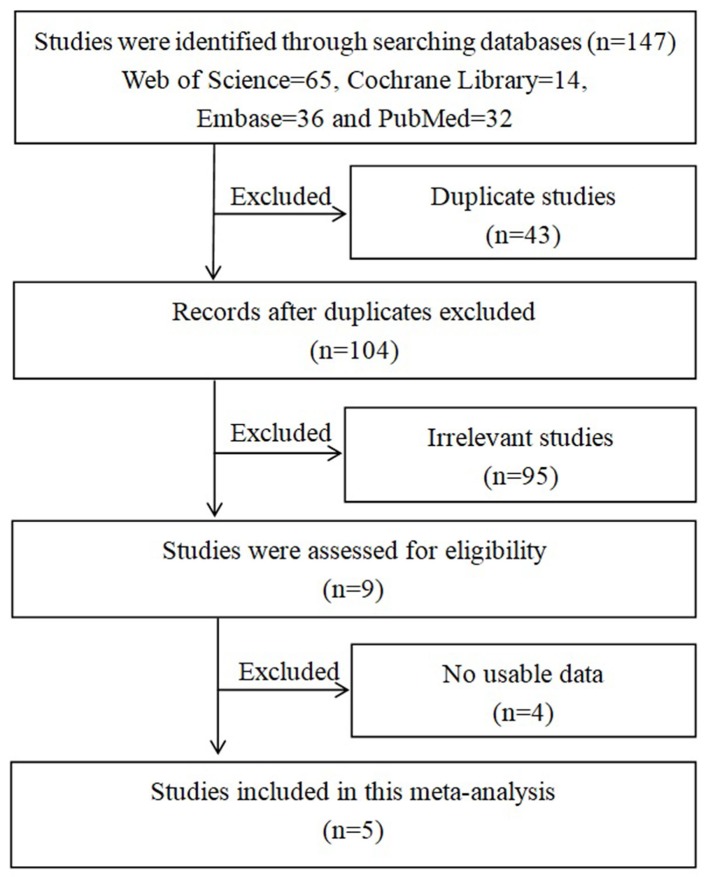
Search flow diagram for this meta-analysis

First, 147 studies were identified through databases searching. Second, 43 duplicate studies were excluded and 95 irrelevant studies were removed by reading titles and abstracts. Finally, 5 studies [18–22] were included in this meta-analysis after excluding 4 studies (Figure 1).

### Characteristics of included studies

The characteristics of included studies are displayed in Table 1. Five studies involving 5533 patients were included in this meta-analysis. These included studies were published from 2016 to 2017 and all from South-Eastern China. The sample sizes ranged from 148 to 2685 in these studies. Three studies focussed on the non-metastatic NPC stage and others based on the metastatic stage or both metastatic and non-metastatic stage. The treatments for patients with NPC were diverse in radiotherapy, chemotherapy, and chemoradiotherapy. The studies’ optimal cut-off values for CRP/Alb ratio ranged from 0.030 to 0.189. Amongst the five studies, all offered OS results, two offered distant metastasis-free survival (DMFS) results and one offered locoregional recurrence-free survival (LRRFS) results. All these included studies were of good quality with the NOS scores ≥7.

**Table 1:** Characteristics of studies included in this meta-analysis

Study	Year	Country	Sample size	NPC stage	Treatment	Cut-off value for CRP/Alb	Outcome	HR with 95% CI	Median follow-up (months)	NOS score
Li et al. [18]	2016	China	409	Non-metastatic/metastatic	R/C+R	0.030	OS	2.093 (1.222–3.587)	53.7 (OS)	7
He et al. [19]	2016	China	2685	Non-metastatic	R/C+R	0.064	OS	1.360 (1.111–1.654)	46.3	7
							DMFS	1.187 (1.012–1.391)		
							LRRFS	1.230 (1.028–1.472)		
Zhang et al. [20]	2016	China	1572	Non-metastatic	R/C+R	0.050	OS	1.394 (1.004–1.937)	50.0	8
							DMFS	1.545 (1.124–2.425)		
Tao et al. [21]	2016	China	719	Non-metastatic	R/C+R	0.141	OS	2.173 (1.128–3.059)	47.0 (OS)	7
Sun et al. [22]	2017	China	148	Metastatic	C	0.189	OS	1.867 (1.085–3.210)	21.8 (OS)	8

Abbreviations: C, chemotherapy; R, radiotherapy.

### Meta-analysis

**Figure 2:**
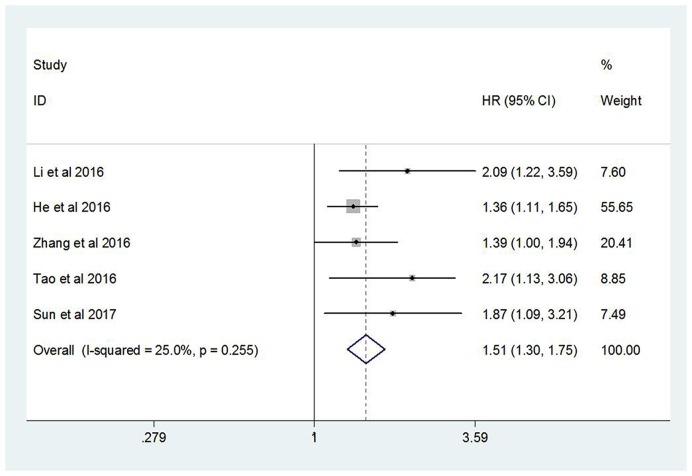
Forest plot of the relationship between CRP/Alb ratio and OS

All these five studies investigated the relationship between CRP/Alb ratio and OS. Because no significant heterogeneity was found (*I^2^* = 25.0%, *P*=0.255), a fixed-effect model was used. The pooled HR was 1.51 (95% CI: 1.30–1.75, *P*<0.001) and revealed that high CRP/Alb ratio was significantly correlated with poor OS, which implied a lower OS rate for the high CRP/Alb ratio group (Figure 2).

**Figure 3:**
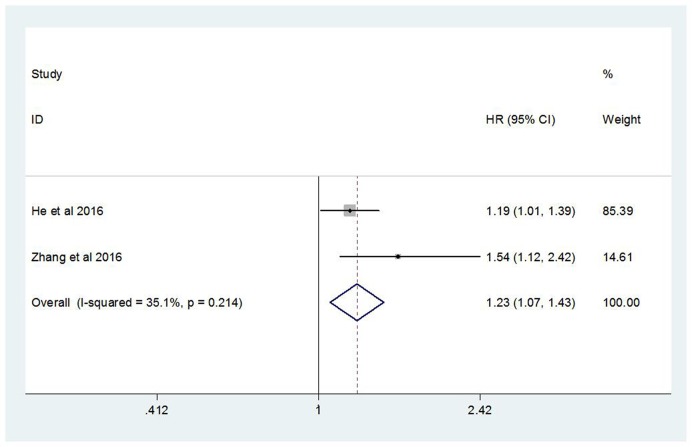
Forest plot of the relation between CRP/Alb ratio and DMFS

There were two studies evaluating the relation between CRP/Alb ratio and DMFS. With no obvious heterogeneity appeared in these two studies (*I^2^* = 35.1%, *P* = 0.214), a fixed-effect model was applied. The pooled HR showed that high CRP/Alb ratio was associated with poor DMFS (HR = 1.23, 95% CI: 1.07–1.43, *P*=0.005). This result also implied that high CRP/Alb ratio group might have higher distant metastasis rate (Figure 3).

### Subgroup analysis

As shown in Table 2, we conducted the subgroup analysis of OS by the sample size and the cut-off value for CRP/Alb ratio. As for these divided subgroups, pooled results showed that high CRP/Alb ratio was related to poor OS (*P*<0.001) and no significant heterogeneity was found. When divided by the cut-off value, higher cut-off value of CRP/Alb ratio had higher HR for OS.

**Table 2:** Subgroup analysis of OS

Subgroups	Number of studies	Number of patients	Pooled HR with 95% CI	*P-*value	Heterogeneity	
					*I^2^* (%)	*P-*value
Sample size						
≤1000	3	1276	2.048 (1.512–2.773)	0.000	0.0	0.917
>1000	2	4257	1.369 (1.155–1.623)	0.000	0.0	0.900
Cut-off value for CRP/Alb						
≤0.1	3	4666	1.423 (1.210–1.674)	0.000	8.5	0.335
>0.1	2	867	2.027 (1.404–2.926)	0.000	0.0	0.686

### Publication bias and sensitivity analysis

Both Begg’s and Egger’s tests were performed to evaluate publication bias in this meta-analysis. When using Begg’s test, no publication bias was found with OS (*P*=0.462). However, publication bias was found with OS (*P*=0.027) when tested by Egger’s test in this meta-analysis. Because the statistic power is relative lower when the enrolled studies were less than ten, we employed the trim and fill method to evaluate the stability of the pooled results [23]. The pooled HR (1.41, 95% CI: 1.23–1.62, *P*<0.001) of OS changed little after performing the trim and fill method. This result demonstrated the stability and robustness of our analysis.

**Figure 4:**
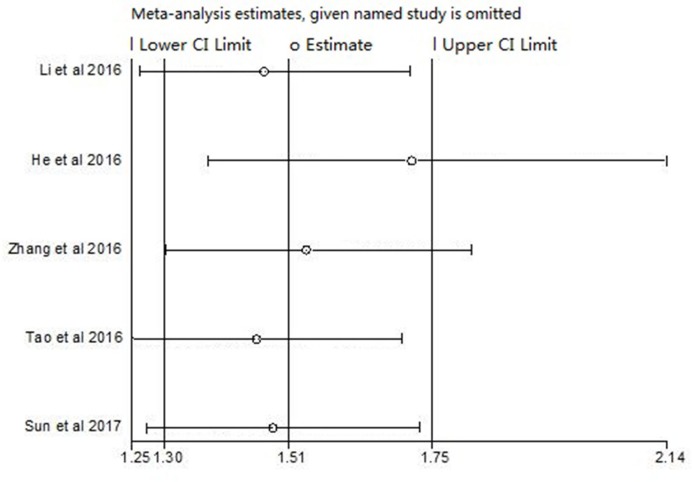
Sensitivity analysis of OS

Sensitivity analysis was used to check whether each study had influence on the pooled HR of OS. Results showed that any individual studies had little effect on pooled results (Figure 4).

## Discussion and conclusion

NPC is an uncommon carcinoma but still a social health problem in the world. Though radiotherapy or chemoradiotherapy is applied in routine treatment and has been improved, the treatment failure rates range from 7% up to 58% [24], which indicates that the outcome of NPC patients is still poor. If we can find some easy-to-get and effective biomarkers to stratify patients and give them optimal treatments, the outcomes may get better.

There are clues that inflammation can enhance tumorigenesis and progression by affecting the microenvironment of cancers [25]. The cancer-related inflammatory response can release some mediators in the microenvironment to promote the development of cancers [26]. The mediators involved in this inflammatory response include cytokines, chemokines, small inflammatory proteins, immune cells, and acute-phase proteins [5]. These mediators may contribute to promoting cancer cells growth, resisting cells death and apoptosis, facilitating angiogenesis, enhancing cells invasion ability, and augmenting metastasis [27,28].

CRP is one of the acute-phase proteins, which is synthesized in the liver and induced by pro-inflammatory cytokines, particularly interleukin-6 (IL-6) [29]. The fact that CRP works as a potential prognostic marker for cancers has been concluded in many researches [30–32]. A meta-analysis with five studies supported the idea that high levels of serum CRP in NPC patients are associated with poor prognosis [33]. Alb is abundant in the serum as a nutritional indicator and an acute-phase protein, which is also involved in the inflammatory response [34]. Its synthesis is stimulated by hormones, while it is inhibited by pro-inflammatory substances, including IL-6 [35]. It also has prognostic value in endometrial cancer [36], ovarian cancer [37], renal cell carcinoma [38], and glioblastoma [39]. Combining the elevated level of CRP (>10 mg/l) and hypoalbuminemia (Alb < 35 g/l), the Glasgow Prognostic Score (GPS) and the modified GPS (mGPS) are prognostic scores for cancers [40–42]. However, most patients with NPC have good performance status and few patients with NPC have hypoalbuminemia before treatment. Therefore, these two prognostic scores may not clarify the prognosis amongst the different NPC stages [21]. Recently, CRP/Alb ratio, a novel prognostic marker, is formed by CRP and Alb and used in many cancers [6–12]. It is a continuous variable, so that it can reflect the inflammation and nutritional status of patients with different stages of NPC. This ratio has a more comprehensive prognostic effect than the single use of CRP or/and Alb.

In the present paper, we performed a meta-analysis to investigate the prognostic role of the pretreatment CRP/Alb ratio in patients with NPC. We included five studies with 5533 patients with NPC. The pooled HR showed that high CRP/Alb ratio was associated with poor OS (*P*<0.001), indicating a lower OS rate for the patients with high CRP/Alb ratio. Then, we analyzed two of these studies which evaluated the relation between the CRP/Alb ratio and DMFS. The pooled HR showed that high CRP/Alb ratio was related with poor DMFS (*P*=0.005). There was no significant heterogeneity between the included studies in this meta-analysis for OS and DMFS. We further conducted the subgroup analysis of OS to confirm the above results and excavate more information for future treatment strategies. When divided by the sample size, pooled HRs showed that high CRP/Alb ratio was related to poor OS (*P*<0.001) in both two subgroups and no significant heterogeneity was found. This result confirmed the prognostic value of CRP/Alb ratio in patients with NPC. When divided by the cut-off value for CRP/Alb ratio, the same results were found with no significant heterogeneity and pooled HRs indicated that the higher cut-off value of CRP/Alb ratio had the higher HR for OS. Based on this result, we hypothesized that in hospitals with higher cut-off value for CRP/Alb ratio, patients with NPC with high CRP/Alb ratio were at greater risk. Moreover, we suggested that hospitals have higher cut-off value for CRP/Alb ratio should pay more attention to their patients with NPC and provide timely treatment in case the patients have worse prognosis. To sum up, elevated CRP/Alb ratio is a useful prognostic marker for patients with NPC.

This meta-analysis has several advantages: first, our meta-analysis added value to the current evidence by providing a large number of samples. Second, no significant heterogeneity amongst the included studies strengthened the results, which also provided a more convincing confirmation for the clinical application of CRP/Alb ratio. Furthermore, the subgroup analysis confirmed the prognostic value of CRP/Alb ratio in patients with NPC and gave the clinical work a suggestion.

There exists some limitations in our meta-analysis. On the one hand, all the included patients were from South-Eastern China. Though the studies concerned a limited geographic and ethnic population, this accorded with the high incidence of NPC in South-Eastern Asia and largely reflected the vulnerable Chinese patients with NPC. On the other hand, NPC patients’ cancer stages, treatment strategies for patients with NPC and follow-up months were diverse, which could have some influence on the pooled results.

In conclusion, our meta-analysis demonstrated that the elevated pretreatment CRP/Alb ratio implied a worse prognosis for patients with NPC. In other words, CRP/Alb ratio is a useful prognostic marker for patients with NPC. Further prospective studies including different populations and larger sample sizes are required to confirm our results.

## Abbreviations

CRP/Alb: C-reactive protein/albumin ratio
DMFS: distant metastasis-free survival
GPS: Glasgow Prognostic Score
HR: hazard ratio
IL-6: interleukin-6
NOS: Newcastle–Ottawa scale
NPC: nasopharyngeal carcinoma
OS: overall survival
95% CI: 95% confidence interval
